# Digital Electrocardiographic Complex for Risk Stratification of Paroxysmal Atrial Fibrillation

**DOI:** 10.17691/stm2024.16.3.05

**Published:** 2024-06-28

**Authors:** A.V. Frolov, O.P. Melnikova, A.P. Vorobiev, T.G. Vaikhanskaya

**Affiliations:** DSc, Professor, Head of the Laboratory of Medical Information Technologies; Republican Scientific and Practical Centre “Cardiology”, Ministry of Health of the Republic of Belarus, 110 R. Luxembourg St., Minsk, 220036, Belarus; Senior Researcher, Laboratory of Medical Information Technologies; Republican Scientific and Practical Centre “Cardiology”, Ministry of Health of the Republic of Belarus, 110 R. Luxembourg St., Minsk, 220036, Belarus; Senior Researcher, Laboratory of Medical Information Technologies; Republican Scientific and Practical Centre “Cardiology”, Ministry of Health of the Republic of Belarus, 110 R. Luxembourg St., Minsk, 220036, Belarus; MD, PhD, Leading Researcher, Laboratory of Medical Information Technologies; Republican Scientific and Practical Centre “Cardiology”, Ministry of Health of the Republic of Belarus, 110 R. Luxembourg St., Minsk, 220036, Belarus

**Keywords:** atrial fibrillation, digital electrocardiography, sinus heart rhythm, heart rate control, “Intecard 8.1”

## Abstract

**The aim of the study** was to develop and clinically test a hardware and software system capable of identifying the predictors of the hidden forms of atrial fibrillation (AF) using 12-lead ECG data in sinus rhythm.

**Materials and Methods:**

There was developed the hardware and software system “Intecard 8.1” to assess a set of markers for atrial electrical instability by 3–5-minute ECG recordings in sinus rhythm. The markers include P-wave amplitude in lead II <0.1 mV, P-wave duration >120 ms, advanced interatrial block, the area of the biphasic P-wave terminal part <–4 mV·ms, and MVP (morphology–voltage– P-wave duration) score >3 points.

The clinical testing of “Intecard 8.1” system was carried out on 120 patients with ischemic heart disease or dilated cardiomyopathy. The patients’ average age was 57.9±13.1 years.

**Results:**

P-wave detection is a challenging task due to a low signal amplitude, noise, high error probability in atrioventricular block or T-wave and P-wave superposition in case of marked tachycardia. To improve detection, a phase transformation method was used, according to which there was studied its phase component arctg[*x*(*n*)*/Rv*], where *x*(*n*) *—* ECG signal samples, *Rv —* a constant. We developed an identification algorithm implemented in “Intecard 8.1” software, its clinical trials being conducted.

During the 12 [6; 22] month observation period, AF episodes were recorded in 22 from 120 patients (18.3%). The patients with AF episodes exhibited a significant decrease in P-wave amplitude (p=0.029), its duration increase (p<0.001), and a significantly high MVP score (p<0.01). The MVP score with a cut-off point >3 points is of the highest prognostic significance. The area under the ROC curve AUC was 0.988 with a 95% confidence interval: 0.975–0.999 (p<0.001). The prediction model of hidden AF paroxysms has sensitivity and specificity: 92 and 89%, respectively.

**Conclusion:**

The digital electrocardiographic complex “Intecard 8.1” when analyzing 3–5-minute ECG recordings with sinus rhythm enables to identify the patients with high risk or with hidden AF forms. The dynamic assessment of P-wave parameters offers an opportunity to personalize heart rhythm control in this patient cohort.

## Introduction

Atrial fibrillation (AF) is the most common form of heart rhythm disorder. The prevalence rate in general population reaches 1–2% with a pronounced growing tendency up to 15% in the senior age group [1]. AF is a confounding factor in arterial hypertension, ischemic heart disease, heart failure, cardiomyopathy, cardiac valve dysfunction, and diabetes mellitus, contributes to these diseases progressing, and aggravates their complications. The risk of cerebrovascular accident in AF increases fivefold [2].

A classic AF diagnostic criterion is the lack of Р-waves on a surface ECG in irregular heart rhythm. However, hidden (asymptomatic) AF forms, their part increasing 20%, are extremely difficult to diagnose. It relates to the fact that routine ECG examinations of most patients in medical facilities show normal sinus heart rhythm [3]. Thus, only a part of AF problem, i.e. the tip of the iceberg, is visible, and comes to the attention of clinical medicine.

There are alternative methods to detect hidden AF. The first one is a long-term monitoring using implanted ECG recorders. Another variant is to reveal the predictor of paroxysmal AF according to ECG findings in sinus heart rhythm. The latter technique is considered to be more available and economically feasible [4].

Sinus rhythm failure in paroxysmal AF occurs due to atrial depolarization dysfunction, due to their conduction delay, the presence of fibrous tissue and rotors, i.e. heterogeneity [5]. The heterogeneity phenomenon, or, in other words, electrical instability of the myocardium, is successfully used in clinical practice: ranging from myocardial ischemia diagnosis to risk stratification of sudden cardiac death [6].

The prognostic value of ECG markers of electrical instability of the atria has been proven by some studies [7–9]. However, the difficulty in distinguishing P-wave parameters on an ordinary electrocardiograph due to its low power and noise-contaminated signal, as well as labor-intensive manual ECG interpretation, hinders their clinical use.

**The aim of the study** was to develop and clinically test a hardware and software system capable of identifying the predictors of the hidden forms of atrial fibrillation (AF) using 12-lead ECG data in sinus rhythm.

## Materials and Methods

There was developed a hardware and software system “Intecard 8.1”, which by precision digital processing assesses a complex of electrical instability markers of an atrial phase of cardiac cycle according to 3–5-minute recordings of ECG signal in 12 standard leads in sinus rhythm.

The complex is based on 12-channel digital ECG communicator and PC computer with a laser printer. Signal measurement range is from 0.03 to 5.0 mV, input impedance — >10 MΩ, in-phase rejection coefficient — 110 dB, response time — >3.2 s, sampling rate — 1000 Hz/channel, bit capacity — 24 bits, output interface — USB 2.0. At the preliminary ECG processing stage we used a set of adaptive digital filters of power line (50 Hz), muscular (35 Hz) and respiratory disturbances (<0.3 Hz), the native signal distortion coefficient being <5% [10]. An electronic magnifying glass with P-wave amplitude gaining up to 30–40 mm/mV was used for visual doctorʼs control.

There were assessed ECG markers of electrical instability of the atria. Among them there were recorded: low amplitude — Р_a_<0.1 mV in lead II, and increased duration of P-wave — P_d_>120 ms [11]; advanced interatrial block at P_d_>120 ms and biphase form of P-wave in leads II, III, and avF [12, 13]; the terminal part area of biphase P-wave <–4 mV·ms in lead V1 [14]; MVP score (morphology–voltage–P-wave duration) >2 points [15].

To assess the prognostic efficiency of “Intecard 8.1” there was carried out testing on a group of 120 patients (mean age: 57.9±13.1 years) with arterial hypertension or dilated cardiomyopathy; among them there were 66.7% males; functional class — I–III according to NYHA; the left ventricular ejection fraction — 45 [40; 51]%. Two-chamber frequency-adaptive cardiac pacemakers made by Medtronic (USA) were implanted to 37 patients (30.8%). The follow-up period was 12 [6; 22] months. Within the mentioned period the patients were recorded AF episodes in 24-hour ECG monitoring or cardiac pacemaker interrogation.

The study was carried out in accordance of Declaration of Helsinki (2013), and approved by the Ethics Committee of Republican Scientific and Practical Centre “Cardiology” (Republic of Belarus). Each patient gave written informed consent.

### Statistical analysis

The results were processed using application packages Statistica 10.0 (Stat Soft) and SPSS Statistics 23.0 (IBM). The findings were represented in the form of M±SD or Me [Q25; Q75] depending on a distribution type. The normality of distribution was determined using Shapiro–Wilk W test. Student t-test, Pearson criterion χ^2^ or Mann–Whitney U test were applied depending on the type of distribution to analyze the differences between the groups. When testing null-hypothesis, a critical significance value was considered equal 0.05. ROC analysis was used to estimate the diagnostic value of the parameters under study. The area under ROC curve (AUC) was accompanied by calculating 95% confidence interval (CI). The null hypothesis in relation to AUC was considered its value equal to 0.5.

## Results

The key point in AF diagnosis is to reveal the fact of P-wave presence or absence, which a cardiologist can notice with the naked eye, especially when using analog ECG recorders. To detect P-wave is the most difficult due to low power signal and high probability to look it over in atrioventricular block or the superposition of Т-wave and P-wave in marked tachycardia.

To identify a low power P-wave there was used the phasor transformation (PT), using which a signal was nonlinearly enhanced. For this purpose discrete sampling of ECG signal *x*(*n*) were represented as *y*(*n*)*=Rv+jx*(*n*), where *Rv* — constant, *j* — imaginary unit. Then we analyzed a signal phase component calculated according to the formula [16, 17]:

φ(n)=arctg[x(n)Rv],

where φ(*n*) *—* signal phase, arctg — arctangent.

**Figure 1.**
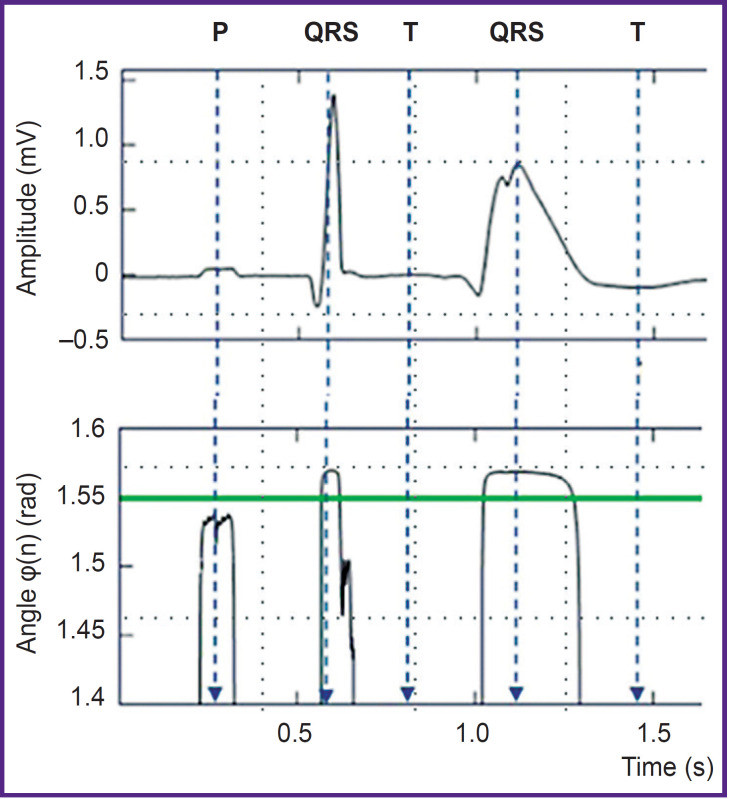
Electrocardiogram (above) and its phasor transformation (below) represented in the form of a phase module |φ(n)| The diagram shows an ordinary cardiac beat with clear P-wave and the following ectopic cardiac beat (ventricular extrasystole) with no P-wave (adapted from [14])

The lower the constant *Rv*, the higher the nonlinear signal amplification coefficient. Figure 1 demonstrates the graph of phase module variation |φ(*n*)*|* in the P-wave area; the normal and the following ectopic cardiac beat (ventricular extrasystole). The normal ventricular contraction is preceded by low-voltage P-wave with significantly enhanced phase signal |φ(*n*)|. In ectopic ventricular contraction the phase signal is close to 0, therefore, there is no P-wave in this cardiac beat.

**Figure 2.**
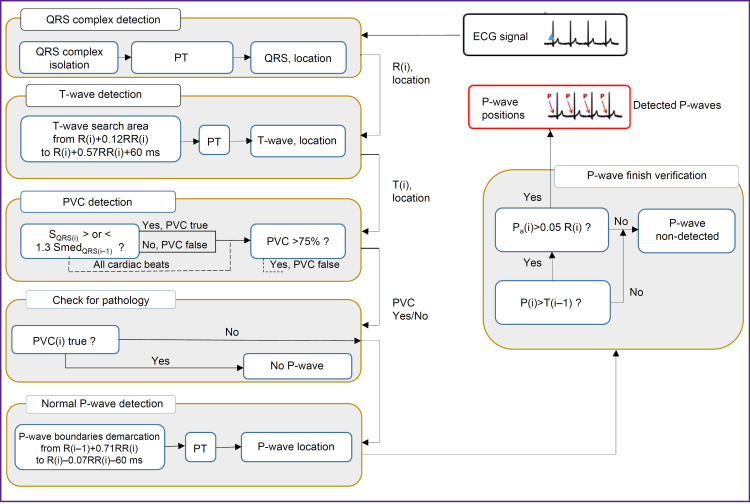
Flow chat of P-wave detection algorithm based on phasor transform Symbols are to be found in the text

There was developed P-wave detection algorithm based on PT method; Figure 2 demonstrates its flow chat. At the first stage there localized QRS complexes, R-waves and RR intervals. Then, in the time window depending on current R(i) and RR(i) values, using PT method, the current T-wave position is located. After that we checked if the current QRS complex belonged to premature ventricular contraction (PVC); for that we compared the area of the current S_QRS(i)_ complex with median area value of the previous Smed_QRS(i–1)_ complexes. S_QRS(i)_ area is calculated in the neighborhood ±150 ms from the current R(i)-wave. If the difference is higher than the threshold value, the current QRS(i) complex is accepted as ectopic PVC, and P-wave in this cardiac beat is not detected. In case the portion of ectopic complexes exceeds 75%, the ECG recording under study is discarded.

Further, P-wave is searched in the absence of PVC complexes. For identification there was used the time window from R(i–1)+0.71RR(i) to R(i)–0.07RR(i)–60 ms, in which applying PT mentioned before, we found maximum φ(*n*) phase, it being taken as a conditional peak of P-wave. The true peak was specified in the neighborhood ±20 ms from the conditional one, by the samples of the previously filtered ECG signal *x*(*n*). In case of biphase P-wave, the peak of the negative phase was found similarly.

P-wave boundaries are identified within the time window specified above. The first out of 20 points from the time window beginning was taken as the start (P_0_), the amplitude of the point being threefold higher than the noise level. Similarly, the end (P_end_) was the first out of 20 points counting from the time window end, the amplitude of the point being threefold higher than the noise level. The least significant bit of an analog-to-digital converter was taken as noise level, in our case — 0.15 μV.

At the last stage two additional checks were carried out: we determined is the current Р(i)-wave went after the preceding Т(i–1)-wave and if amplitude Р(i) exceeded the threshold equal to 0.05 R(i). If the answer is yes, then P-wave is considered detected; otherwise, it is not (see Figure 2; Figure represents no cases of P-wave detection in atrioventricular II stage block, since in this situation there is no QRS complex, as well as in superposition of T-wave and P-wave in marked tachycardia).

Clinical testing of “Intecard 8.1” complex was carried out in a group of 120 patients. Asymptomatic AF episodes (recorded in interrogating cardiac pace maker or in 24-hour ECG monitoring) were taken as the primary endpoint (event). All the patients underwent past history screening, physical examination, and 3–5-minute ECG recording in 12 standard leads, with P-wave parameters assessment using the developed computer program “Intecard 8.1”. The patients also had 24-hour ECG monitoring (Oxford Medilog; Oxford, Great Britain), echocardiography (Vivid 7; General Electric, USA) and biochemical blood assay. A highly experienced cardiologist had control over the correctness of softwarebased evaluation of P-wave parameters.

During the observation period (12 [6; 22] months), 22 out of 120 patients (18.3%) were found to have AF episodes. AF detection rate in patients with or without an implanted cardiac pacemaker was 35.1 and 10.8%, respectively. Table 1 represents the main characteristics of the patients under study.

**T a b l e 1:** Characteristics of patients with and without recorded episodes of atrial fibrillation, M±SD

Parameters	Group without AF episodes (n=98)	Group with AF episodes (n=22)	p
Age (years)	57.4±14.8	59.9±10.7	0.658
Body mass index	29.6±5.6	29.8±7.0	0.734
Ejection fraction (%)	53.1±9.7	43.3±9.7	0.045
Left atrium size (mm)	45.2±5.6	44.9±5.9	0.631
CHA_2_DS_2_-VASc scale (points)	1.6±1.2	1.6±1.2	0.989
PR interval (ms)	182±40	185±46	0.813
P-wave amplitude (mV)	0.16±0.02	0.09±0.03	0.029
P-wave duration (ms)	112±12	146±16	<0.001
MVP score (points)	2.0±1.0	3.8±1.1	<0.01
Advanced interatrial block (%)	53.1±9.7	43.3±9.7	0.045
Terminal part area of biphase P-wave (mV·ms)	-3.3±3.9	-4.5±4.2	0.439

When comparing the groups, there were no significant differences in age, risk scale CHA_2_DS_2_-VASc, the left atrial size, and PR intervals. In addition, the patients with AF episodes had significantly decreased P-wave amplitude: 0.09±0.03 vs. 0.16± 0.02 mV (p=0.029); its duration increased: 146±16 vs. 112±12 ms (p<0.001); significantly high MVP score was recorded: 3.8±1.1 vs. 2.0±1.0 points (p<0.01). Advanced interatrial block rate appeared to be slightly higher in patients without AF episodes: 53.1±9.7 vs. 43.3±9.7% (p=0.045). No significant differences were revealed when estimating the terminal part area of biphase P-wave: –3.3±3.9 vs. –4.5±4.2 mV·ms (p=0.439).

Table 2 demonstrates the assessment results of AUC areas for AF ECG predictors and stroke risk scales CHA_2_DS_2_-VASc.

**T a b l e 2:** AUC area assessment results for ECG predictors of atrial fibrillation

Parameters	Cut-off point	AUC	95% CI	p
MVP score (points)	3	0.988	0.975–0.999	<0.001
P-wave duration (ms)	130	0.878	0.777–0.979	<0.01
Scale CHA_2_DS_2_-VASc (points)	3	0.512	0.372–0.652	0.989

MVP score (morphology-voltage-P-wave duration) with the cut-off point >3 points has the highest prognostic value; the area under ROC curve AUC was 0.988 in 95% CI: 0.975–0.999 (p<0.001). P-wave duration >130 ms appeared to be a significant predictor as well; AUC=0.878 in 95% CI: 0.777–0.979 (p<0.01). Risk scale CHA_2_DS_2_-VASc >3 points revealed no prognostic characteristics. AUC area was just 0.512 in 95% CI: 0.372–0.652 (p=0.989). Since MVP score is an integrated parameter including P-wave duration as well, the parameter was the only one involved in the prognostic model; the parameter sensitivity and specificity were 92 and 89%, respectively.

Thus, MVP score integrating pathological morphology, amplitude and duration of P-wave was defined as a basic independent predictor of paroxysmal AF in a study group of patients with ischemic heart disease and dilated cardiomyopathy.

## Discussion

Clinical trials of hardware and software system “Intecard 8.1” showed the feasibility to acquire valuable prognostic information from ECG concerning the probability of paroxysmal AF in patients, if there were no AF diagnostic signs during the current ECG examination. The present study stated 3–5-minute ECG recordings in sinus rhythm containing the marker of electric instability to enable to identify the patients with high risk of AF or those with previous overlooked paroxysmal AF. Currently, widely used risk scales APPLE, BASE-AF, CHA_2_DS_2_-VASс, LOGO and others have low prognostic accuracy, since they are only based on population factors such as age, gender, CHD, arterial hypertension, type 2 diabetes in their past history. In assessment methods mentioned above the area under ROC curve AUC is within the range from 0.55 to 0.67 [18]. Owning to inadequate accuracy of AF risk scales, clinicians frequently run into difficulties when choosing optimal treatment for each patient [19]. Our findings comply with those facts stated before.

The use of information on electrical atrial instability was found to improve the accuracy of an individual prognosis. For example, the study by Kreimer et al. [20] demonstrated predictive ability of low-voltage P-wave, advanced interatrial block, terminal part of biphase P-wave and MVP score. In patients with no predictors mentioned, AF risk was just 4%, with one predictor — 18%, with two predictors — 41%, and with four — 70% (p<0.01) [20]. Our results revealed a dominating prognostic role of MVP score combining the morphology change, voltage decrease, and P-wave extension. P-wave duration of more than 130 ms demonstrates the conductivity slowdown due to the replacement of myocardium by fibrous tissue, and MVP score >3 points indicates the left ventricular remodeling [9].

To solve the problem of detecting hidden cardiovascular pathology it is promising to apply artificial intellect (AI). So, AI improved by 25% AF prognosis accuracy compared to stroke risk scale CHA_2_DS_2_-VASc. However, for successful use of AI it is necessary to integrate considerable volumes of ECG data that can be achieved only as a part of multicenter studies. For instance, Attia et al. studied over 650,000 ECG with AF to develop an AI model of AF prognosis [21].

It should be noted that “Intecard 8.1”, the software we developed, significantly facilitates the measurements of low-observable P-wave parameters on surface ECG. Nevertheless, the current version of the program does not use a complete set of precise parameters of atrial systole. Our near-term plans consist in including into analysis the following: spatial and time dispersion, pathological misalignment of the electric axis, as well as P-wave chaos based on 3–5-minute ECG recordings.

The method limitations include a comparatively small group of patients under study and undetection probability of rare AF paroxysms.

## Conclusion

The digital electrocardiographic complex “Intecard 8.1” according to the analysis of 3–5-minute ECG recordings with sinus rhythm enables to identify high risk patients with atrial fibrillation. The dynamic assessment of P-wave parameters offers an opportunity to personalize heart rhythm control in this patient cohort.

Non-invasiveness, high carrying capacity and relatively low cost of digital electrocardiographic equipment provide availability of the developed technology for all public health facilities including primary health care.
